# A Narrative Review of the Classical and Modern Diagnostic Methods of the No-Reflow Phenomenon

**DOI:** 10.3390/diagnostics12040932

**Published:** 2022-04-08

**Authors:** Larisa Renata Pantea-Roșan, Simona Gabriela Bungau, Andrei-Flavius Radu, Vlad Alin Pantea, Mădălina Ioana Moisi, Cosmin Mihai Vesa, Tapan Behl, Aurelia Cristina Nechifor, Elena Emilia Babes, Manuela Stoicescu, Daniela Gitea, Diana Carina Iovanovici, Cristiana Bustea

**Affiliations:** 1 Department of Medical Disciplines, Faculty of Medicine and Pharmacy, University of Oradea, 410073 Oradea, Romania; larisa.rosan@yahoo.com (L.R.P.-R.); babes.emilia@gmail.com (E.E.B.); manuela_stoicescu@yahoo.com (M.S.); 2 Doctoral School of Biological and Biomedical Sciences, University of Oradea, 410087 Oradea, Romania; diana_iovanovici@yahoo.com; 3 Department of Pharmacy, Faculty of Medicine and Pharmacy, University of Oradea, 410028 Oradea, Romania; gitea_daniela@yahoo.co.uk; 4 Department of Preclinical Disciplines, Faculty of Medicine and Pharmacy of Oradea, University of Oradea, 410073 Oradea, Romania; mada_vidican@yahoo.ro (M.I.M.); v_cosmin_15@yahoo.com (C.M.V.); cristianabustea@yahoo.com (C.B.); 5 Department of Dental Medicine, Faculty of Medicine and Pharmacy, University of Oradea, 410073 Oradea, Romania; panteavladalin@yahoo.ro; 6 Department of Pharmacology, Chitkara College of Pharmacy, Chitkara University, Punjab 140401, India; tapanbehl31@gmail.com; 7 Analytical Chemistry and Environmental Engineering Department, Polytechnic University of Bucharest, 011061 Bucharest, Romania; aureliacristinanechifor@gmail.com

**Keywords:** diagnostic methods, no-reflow phenomenon, electrocardiogram, echocardiogram, thrombolysis, acute myocardial infarction, myocardial blush grade

## Abstract

The incidence of the no-reflow (NR) phenomenon varies depending on the diagnostic criteria used. If just the angiographic criteria are considered (i.e., a degree of thrombolysis in myocardial infarction ≤2), it will be found that the incidence of NR is quite low; on the other hand, when the myocardial NR is taken into account (i.e., a decrease in the quality of myocardial reperfusion expressed by the degree of myocardial blush), the real incidence is higher. Thus, the early establishment of a diagnosis of NR and the administration of specific treatment can lead to its reversibility. Otherwise, regardless of the follow-up period, patients with NR have a poor prognosis. In the present work, we offer a comprehensive perspective on diagnostic tools for NR detection, for improving the global management of patients with arterial microvasculature damage, which is a topic of major interest in the cardiology field, due to its complexity and its link with severe clinical outcomes.

## 1. Introduction

The no-reflow (NR) phenomenon is highlighted when, following a myocardial infarction (MI), after the efficient unclogging of the epicardial coronary artery involved in the infarction, inadequate myocardial infusion is observed. Although effective methods of vascular permeabilization (such as coronary angiography) are currently available, the incidence of the phenomenon remains high, being found in 0.6 to 3.2% of patients who have had percutaneous coronary intervention (PCI) [1,2,3].

Coronary NR, detected angiographically, occurs when the degree of thrombolysis in myocardial infarction (TIMI) flow and the myocardial blush grade (MBG) is ≤2, and has been found in 2% of patients who underwent interventional myocardial revascularization. The two types of NR are as follows:

- a NR phenomenon identified by angiography, expressing the degree of TIMI and MBG flow;
- a myocardial NR that may occur in patients who have obtained excellent resumption of intracoronary flow, but in whom the quality of reperfusion in the microcirculation is poor [4].

Estimation of the incidence of the phenomenon evaluated only according to angiographic criteria underestimates its presence, because these instances are limited only to coronary NR. However, taking into account the quality of myocardial reperfusion (and therefore myocardial NR), there is a significant increase in the incidence of this phenomenon, which can be found in a third of patients with acute MI (AMI) [5].

The illusion of myocardial reperfusion following myocardial revascularization, as well as the prognosis of these patients, is of real interest in assessing the quality of coronary microcirculation reperfusion, beyond simply assessing the resumption of optimal epicardial coronary flow. In order to achieve a reliable assessment of the quality of coronary reperfusion, several imaging techniques are available to cardiologists: post-stent angiographic evaluation, electrocardiogram (ECG), and more recently new imaging techniques including myocardial contrast echocardiography (MCE), cardiac magnetic resonance imaging (CMRI), and positron emission tomography (PET) [6].

NR becomes more frequent as the duration of myocardial ischemia becomes longer. At the myocardial level, NR does not occur unless the reperfusion of the coronary artery responsible occurs before the installation of irreversible myocardial lesions (such as the appearance of myocardial necrosis in ischemic territory). As a consequence, after the onset of myocardial necrosis, the phenomenon of NR is expected to occur [7].

A dynamic process can evolve after myocardial reperfusion, worsening the ischemic lesions. The prognosis of patients with NR is unfavorable, as they are prone in the short term to the occurrence of malignant rhythm disorders and sudden cardiac death, and in the long term to the occurrence of heart failure. Recently, there have been significant improvements in technique and pharmacotherapy that have significantly changed the prognosis of patients [8]. Published experimental studies have revealed the relationship between NR and demographic, biochemical, and anatomical parameters. According to the results obtained, female gender, advanced age, diabetes mellitus, multi-vessel involvement, and delayed reperfusion can all predict the probability of NR following primary PCI (PPCI) in the setting of AMI [9].

Despite significant advancements in the prevention and treatment of cardiovascular disorders, women continue to be underdiagnosed and undertreated, with greater rates of hospitalization and death than males [10].

Due to the multifactorial nature of the phenomenon, different therapy approaches are required. The use of vasodilators such as verapamil, adenosine, nitrates, nicardipine, sodium nitroprusside and papaverine is common in today’s pharmacological therapy; however, a vasoconstrictor-like epinephrine may also play a significant role [11]. Furthermore, polyphenols may have a beneficial role due to their pleotropic effects on the cardiovascular system [12].

Poor cardiovascular outcomes are the leading cause of death in many nations, responsible for almost one-third of all deaths worldwide. More effective diagnostic methods and non-invasive imaging approaches are essential in providing detailed information about different pathologic conditions. Nanotechnology contains nanoscale structures with unique physicochemical features that make them desirable for improving present diagnosis procedures, and may play a beneficial role in diagnosing NR in conjunction with imaging techniques [13].

Detection of NR after PPCI in patients with segment elevation myocardial infarction (STEMI) is essential because it represents the failure of myocardial reperfusion, despite recanalization of the infarcted epicardial coronary artery. Although the incidence of NR today is low, many of these patients have not been diagnosed correctly, and the prognosis is unfavorable, both in the short and long term, compared to those who do not develop the pathology [5].

In the aforementioned context, this narrative review highlights the updates available for NR diagnosis and treatment in patients with STEMI, a phenomenon often ignored or underestimated, although there are many diagnostic methods available to specialists in the field. Our study also provides a very well documented pathogenesis of NR and a relevant and necessary update in the field of NR diagnostics methods, very useful for cardiologists and other medical specialists.

## 2. Methods

**Figure 1:**
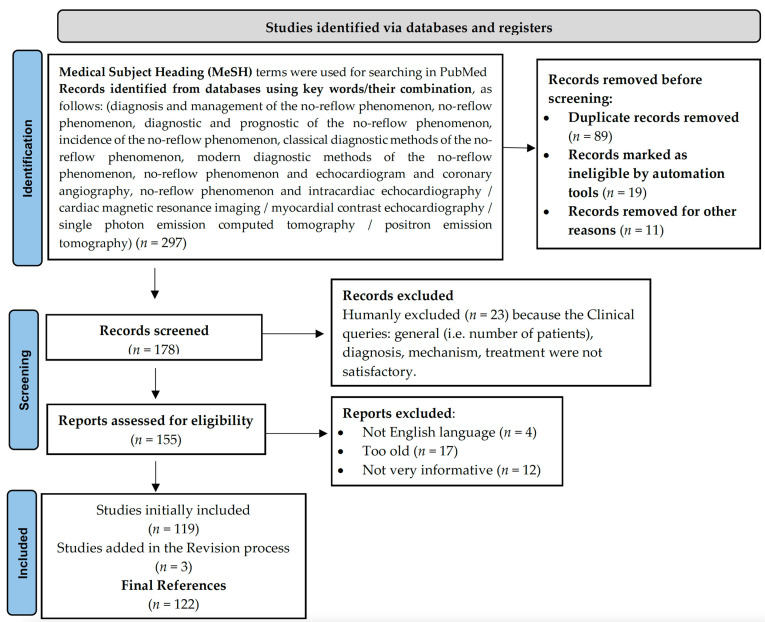
PRISMA 2020 flow diagram describing literature selection.

This paper identified and filtered academic publications focusing on the NR phenomenon between 1992–2022, following a comprehensive search of the literature (the main platform for the key word search was PubMed) in the topic of the management of NR, highlighting and detailing the most important diagnostic methods available for use in the field of cardiology (Figure 1, according to Page et al.) [14].

## 3. Pathogenesis of the No-Reflow Phenomenon

The NR phenomenon occurs in patients with AMI, in whom after myocardial revascularization, either by PPCI or thrombolysis, there is an obstruction in the microcirculation, resulting in failure of revascularization of the infarcted area and correlating with a poor prognosis in these patients [8,15]. Most studies regarding NR in AMI patients undergoing PCI were limited to single-center studies. Table 1 shows the incidence of NR correlated with different diagnostic methods.

The disorders of the coronary microcirculation that induce NR are determined by five primary elements: pre-existing microcirculation lesions, distal microembolization, ischemic myocardial injury, myocardial reperfusion injury, and individual susceptibility [26].

### 3.1. Pre-Existing Lesions of the Microcirculation

Pre-existing damage to the microcirculation may be structural/organic, functional, or both. The existence of microcirculation dysfunction affects myocardial revascularization and restricts the benefits of coronary reperfusion, increasing the vulnerability of the affected myocardium to injury that may be induced by PCI [27,28].

Organic microcirculation injury is rendered by combining three factors, i.e., myocytes in the necrotic area, coronary artery endothelium and blood element figures. Myocytes that are contained in the infarcted area become swollen and may end up compressing the endothelium of the capillaries, especially when interstitial oedema is present in the infarct area. Endothelial cells protrude into the vascular lumen due to their oedema, which can obstruct the capillary lumen. At the same time, fibrin-platelet microthrombi may be observed at this level, which may be formed by platelet activation induced by endothelial injury, or may be produced due to embolized micro-fragments from the thrombus responsible for the infarct. On the other hand, leukocytes may also obstruct the capillary lumen, activated and attracted by local ischemia. Once inside the vascular lumen, they can interact with platelets via free radicals, leukotrienes and proteolytic enzymes, which ultimately result in morpho-functional alteration of endothelial cells [26].

Functional damage to the microcirculation is observed via a significant reduction in vasodilator output. Macro- and microcirculatory vasoconstriction may also be influenced by adrenergic hypertonia, as well as by increased expression of angiotensin II receptors, or endovascular smooth muscle contracture due to ischemia [28].

### 3.2. Distal Microembolization

Obstruction of the microcirculation is an important element in the occurrence of NR, and may be caused by microthrombi from the thrombus that have occluded the epicardial coronary artery, or thrombotic material from ruptured or fissured atheromatous plaque. Thus, following attempted myocardial revascularization by PCI, depending on the technique used, balloon dilatation or stent implantation, and on the quality of the atheroma plaque and the amount of fibrin present in the thrombotic material, microemboli may detach and obstruct the coronary microcirculation. When more than half of the coronary capillaries are obstructed, myocardial perfusion decreases, with the occurrence of NR. This phenomenon is less common in patients who have undergone myocardial revascularization with thrombolytics [29,30].

### 3.3. Ischemic Myocardial Injury

The occurrence of myocardial ischemia through obstruction of an epicardial coronary artery, common in AMI, induces consecutive ischemia in the capillaries in the area of the ischemia. The capillary wall may show discontinuities in the form of “gaps”, losing its integrity, with the collection of erythrocytes [31].

### 3.4. Myocyte Reperfusion Injury

Following an AMI, reperfusion of the obstructed and thus infarct-causing epicardial coronary artery is essential. However, when myocardial ischemia occurring post-MI has been in place for a prolonged period, exceeding the optimal myocardial revascularization time elapsed from the onset of typical symptoms until PCI is performed, myocardial revascularization may worsen the established endothelial damage. Myocardial reperfusion leads to an increased influx of platelets and neutrophils into the area of ischemia, leading to neutrophil activation with production of vasoconstrictors and inflammatory mediators, ultimately producing nitric oxide, prostacyclin and endothelin, which together with ischemic lesions favor the development of intramyocardial hemorrhage [32,33,34].

### 3.5. Individual Susceptibility

**Figure 2:**
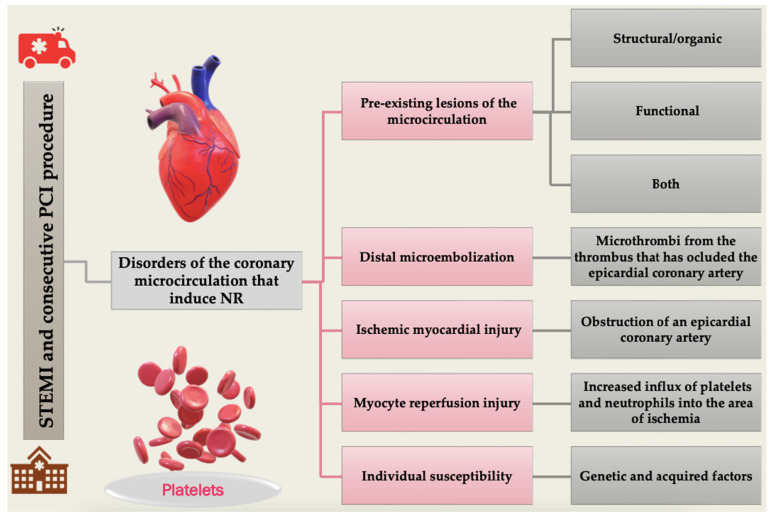
The main pathophysiological mechanisms involved in no-reflow (NR) apparition in STEMI after PCI, mainly occurring because of pre-existing lesions or other favoring factors, or worsened by the procedure itself. PCI, percutaneous coronary intervention; STEMI, segment elevation myocardial infarction.

Individual susceptibility can occur due to genetic or acquired factors. Cardiovascular risk factors may also influence the occurrence of microvascular obstruction (MVO). However, the exact mechanisms have not been fully elucidated and require further research [27,35]. The pathophysiological processes that lead to the appearance of NR phenomenon are summarized in Figure 2.

## 4. Predictors of the No-Reflow Phenomenon

Predictors of NR can be divided into two categories: classical cardiovascular risk factors and angiographically demonstrated predictors. It has been found that NR is more common in patients with several associated cardiovascular risk factors. Thus, patients who are older, obese, dyslipidemic, diabetic, hypertensive, or smokers who developed STEMI are more prone to NR after myocardial revascularization due to pre-existing microvascular endothelial dysfunction [36].

A direct proportional relationship of the involvement of visceral obesity in the development of atherosclerosis has been detected by measuring body mass index, explained mainly by the production of proinflammatory elements and other enzymes with an unfavorable effect on the vascular endothelium [37].

The impact of dyslipidemia is due to the increase in plasma total cholesterol level, which is the main risk factor in triggering the atherogenesis process. The first changes that appear in the arterial wall are prominent yellow lipid streaks on the surface of the vascular endothelium, formed predominantly by foam cells. The next changes are the appearance of fibrous atheromatous plaques which can cause partially or totally occlusive vascular lesions. They may have a fibrous consistency with a lower lipid content, or a soft consistency due to a high lipid content [38,39].

As changes continue, atheroma plaque complication may be observed, including cracking, rupture or ulceration of the atheroma plaque which can lead to the development of local hemorrhaging and thrombosis. Depending on the size of the thrombi, they may partially or totally obstruct the vascular lumen, leading to AMI. Low-density lipoprotein cholesterol indirectly contributes to the instability of atheromatous plaques, carrying an increased atherogenic risk, which will contribute to the development of the vulnerable atheromatous plaque and finally to its rupture, obstructing of the vascular lumen and the development of AMI [40,41,42].

Regarding the role of diabetes mellitus in determining ischemic lesions and the occurrence of NR, it has been observed that both insulin- and non-insulin-dependent diabetes mellitus are associated with an increased risk of ischemic cardiovascular disease [43]. However, diabetes mellitus remains a controversial risk factor, the mechanism of action not being fully elucidated; its involvement in altering lipid metabolism is thought to lead to the development of atherosclerosis [44].

Hypertension is a particularly important risk factor for cardiovascular disease. However, it is also a controversial factor, as hypertension facilitates the action of other factors causing endothelial dysfunction [45].

It has also been observed that following epicardial coronary artery re-permeabilization in the development of AMI by PPCI, NR was more frequently encountered in patients with late presentation to the medical unit where PCI was performed. This is explained by the enlargement of the ischemic zone and the appearance of myocardial necrosis, which leads to the occurrence of the phenomenon. Also, the infarcted anterior territory as well as the anterior descending artery are more frequently incriminated in the occurrence of NR, as well as the implantation of an increased number of stents for revascularization of the involved coronary artery [46,47].

**Figure 3:**
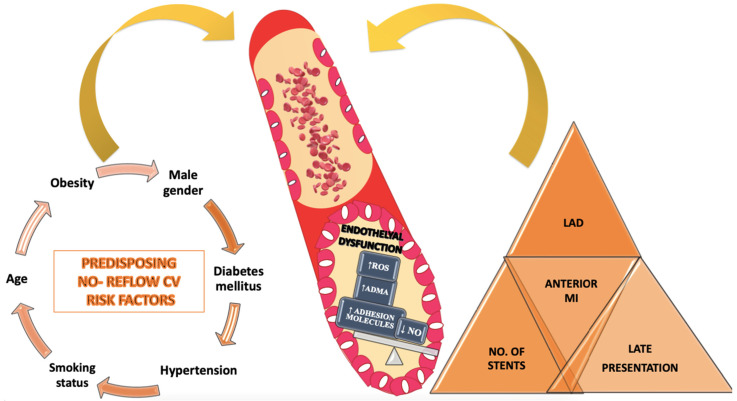
Predictors of the no-reflow phenomenon. ADMA, asymmetric dimethylarginine; CV, Cardiovascular; LAD, left anterior descending coronary artery; MI, myocardial infarction; NO, nitric oxide; ROS, reactive oxygen species.

Regardless of the type of NR phenomenon, its occurrence is determined by multifactorial causes including non-modifiable and modifiable cardiovascular risk factors, as well as the location of STEMI involving the anterior descending artery as the responsible artery, revascularization time, and intracoronary drug management of the coronary NR [48]. The predictors involved in the appearance and evolution of NR are depicted in Figure 3.

## 5. Diagnostic Methods of the No-Reflow Phenomenon

Due to the wide range of current approaches for evaluating the clinical NR phenomenon, it is essential to assess the mechanisms and limitations of the most frequently used diagnostic tools.

### 5.1. Classical Diagnostic Tools for the No-Reflow Phenomenon Detection

There are available several methods available to diagnose NR; we initially consider the classical methods, including electrocardiogram (ECG), coronary angiography, TIMI-flow grade, corrected TIMI frame count (CTFC), MBG and TIMI myocardial perfusion grade (TMPG) [49].

#### 5.1.1. ECG

**Figure 4:**
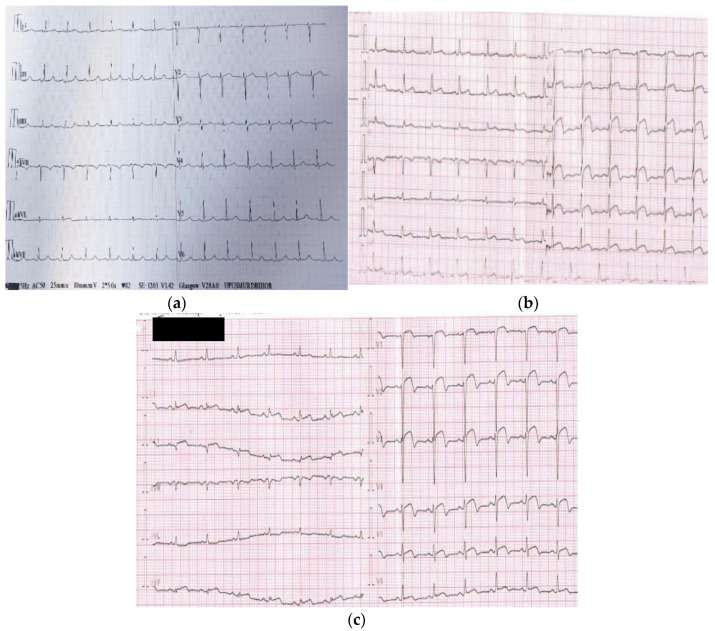
Characteristic aspects of no-reflow phenomenon diagnosis based on ECG: (**a**) normal ECG without ST segment changes (isoelectric); (**b**) ECG in a subject diagnosed with STEMI at admission showing anterior ST segment elevation of 6 mm (precordial leads V1-V6); (**c**) the same patient from (**b**)—ECG at 2 h after PPCI of the culprit lesion showing persistent ST segment elevation of 5–6 mm (precordial leads V1-V6) without expected ST segment resolution of more than 70%.

The ECG is a standard method of assessing the NR process, which is defined as prolonged ST elevation despite opening the epicardial coronary artery in the setting of a STEMI [50,51,52]. Figure 4 (images from the personal medical archive of the first author) highlights the diagnosis of NR phenomenon based on ECG characteristics. Patients with prolonged ST elevation in more than two contiguous leads after reperfusion even though the epicardial blood flow is normal tend to have a higher mortality risk. Early ST-segment resolution is linked to left ventricular ejection fraction (LVEF) and to the extent of an enzymatic infarct [53,54].

Long-term mortality following PPCI is predicted by persistent ST elevation and MBG grades 0 to 1, while CTFC is a weaker predictor. Concomitant use of these features may improve their predictive power [55].

ST resolution can be obtained in simply and quickly; however, in some cases it can be an ineffective tool for diagnosing the NR phenomenon. In a comparative study, incomplete ST resolution was correlated to baseline LV function, but not to changes over time [56]. Moreover, the linking between ST segment alterations and myocardial perfusion as measured by MCE has been assessed in patients with AMI treated with PPCI. After medical intervention, all the patients’ TIMI 3 flow was restored. Although less sensitive, a quick ST segment reduction was highly specific (91%) for myocardial reperfusion [57].

It has been shown that a rapid reduction in ST elevation after reperfusion therapy is correlated with early, complete, and quick restoration of cardiac tissue perfusion. Furthermore, successful tissue perfusion is also associated with early T-wave inversion. Following cardiac reperfusion therapy, sustained elevation of the ST segment is linked with poor clinical and functional results. Thus, ST segment monitoring is an effective and inexpensive approach to assess myocardial reperfusion [58].

#### 5.1.2. Coronary Angiography

Using TIMI flow grades, angiographic and clinical outcomes following PPCI are commonly correlated with thrombolysis. The hypothesis is that contrast fluidity in the epicardial coronary artery represents spontaneous coronary circulation and myocardial perfusion, as well as the efficacy of coronary intervention [59].

NR can be evaluated without additional cost by using simple angiographic procedures at the time of PCI. The CTFC is the number of cine frames between contrast injection and the frame in which contrast reaches a standardized distal marker in the culprit artery, and is generally used to show enhanced therapeutic flow. TIMI flow grade is exclusively sensitive to epicardial blood flow and has low sensitivity to microvascular blockage. The quantity of contrast that passes through and opacifies cardiac tissue is measured by myocardial perfusion grade, and more frequent MBG. MBG b3 was found in 18–37% of patients with normal TIMI grade 3 flow after PPCI for MI [23,60].

Existing medical evidence suggests the use of “angiographic criteria” to identify NR, such as TIMI flow grade b3 or TIMI flow grade 3 plus MBG b3. By TIMI flow grade, the incidence of NR after PPCI was 12–37% and by MBG was 29–63% [61,62,63].

##### TIMI Flow Grade

TIMI flow grades are provided on a scale of 0 to 3. Improved clinical outcomes and decreased mortality have been linked to higher TIMI flow grades. During coronary angiography, TIMI blood flow grades are used to assess the quality of coronary flow [64,65].

**Figure 5:**
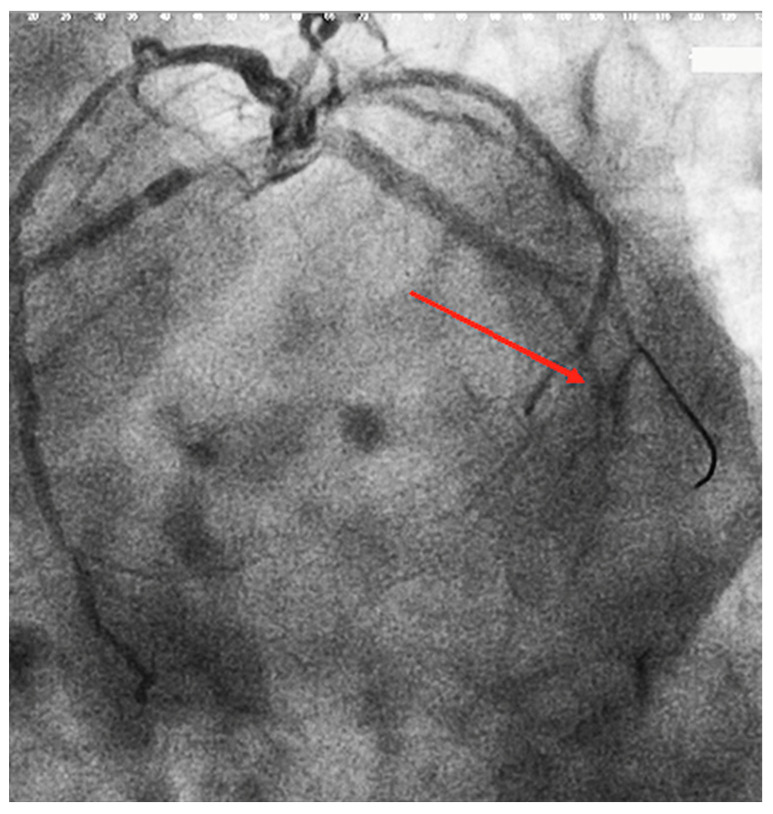
No-reflow phenomenon appear at the circumflex artery (indicated by the red arrow).

This method assesses the clearance of radiographic dye in the coronary arteries. Patients with TIMI 0 or 1 flow are considered to have experienced failed reperfusion (Figure 5—image from the personal medical archive of the first author–shows the appearance of TIMI 0/1 flow after de stent implantation), while those with TIMI 2 or 3 flow are considered to have had an effective reperfusion.

Since TIMI flow grades are not fully accurate, large experimental thrombolysis trials have shown that patients with TIMI flow grades 1 or 2 have similarly poor prognosis to those with TIMI flow grade 3. Thus, a TIMI flow grade < 3 would be accurate enough for diagnosis of MI reperfusion NR [66]. According to results from experimental studies that applied MCE, TIMI flow grade 2 is correlated with a large NR area; consequently, only TIMI flow grade 3 shows reperfusion success [16,67].

Medical data from the TEAM-2 study found that early TIMI flow grade 3 in patients receiving streptokinase was correlated with lower hospital mortality, decreased peaks of cardiac biomarkers, and ECG indicators of MI. Furthermore, patients with TIMI-flow grade 2 had no statistically significant differences in biomarker activity, ECG indicators, or short-term survival, compared to those with grade 0 or 1 [68].

##### CTFC

CTFC is an approach to examine coronary circulation more objectively. CTFC refers to the number of cine frames needed for radiocontrast dye to reach standardized distal markers in the epicardial arteries, adjusted for artery length differences. Lower CTFC correlates with intracoronary Doppler-measured coronary blood flow velocity; nevertheless, while CTFC accurately reflects blood flow, it does not accurately determine the extent of microvascular injury [69].

Even though a faster 90 min CTFC may be associated with improved clinical outcomes for hospitalized patients, it is possible that the observed increase in outcomes was due to a subset of individuals with supranormal CTFC [70,71].

##### MBG

MBG is used to evaluate myocardial staining following PPCI. After radiocontrast injection, the scale indicates the emergence and disappearance of myocardial blush. The original scale was 0 to 3, with 3 representing normal dye entry and exit in the myocardium, similar to what can be visualized in a non-infarct-related epicardial artery; 2, moderate myocardial blush or contrast density, but less than is seen in a non-infarct-related artery; 1, minimal contrast density or blushing; and 0, no blush or persistence of MBG, implying leakage into the extravascular space [72].

Even in patients with TIMI flow grade 3, MBG assessments by the operator during PPCI appear to independently predict 1-year all-cause death. However, recent research has brought into question these earlier conclusions, with neither the TIMI flow grade nor the MBG were found to be connected [73].

According to a post hoc study of the Controlled Abciximab and Device Investigation to Lower Late Angioplasty Complications (CADILLAC) trial, the degree of MBG and the ST-segment resolution 4 h after PPCI are commonly discordant (in around 40% of cases), which may limit their use. ST resolution was a more accurate indicator of clinical outcomes at one month and at one year after multivariable adjustment; however, both factors in combination appeared to have added predictive value [74].

##### TMPG

TMPG, which is similarly rated on a scale of 0 to 3, is another way to assess myocardial perfusion. TMPG grade 3 blush represents the start of blush clearance during washout (i.e., minimally persistent after three cardiac cycles of washout); TMPG grade 2 blush clears mildly or not at all during three cardiac cycles of washout; TMPG grade 1 blush indicates the presence of myocardial blush without clearance from the microvasculature (i.e., stain was present during the next injection); grade 0 blush indicates no perfusion at tissue level [75,76].

TMPG is a densitometric method for grading the evolution of contrast media at the myocardial level (i.e., entrance, persistence, and elimination). To grade NR, it can be combined with the TIMI flow grade [77].

MBG, on the other hand, assesses the intensity of the blush in the distribution of the culprit artery compared to the contrast density in unaffected areas. Independent of epicardial blood flow restoration, impaired reperfusion as defined by the TMPG grading system is linked to a higher risk of mortality [75].

TMPG had the highest correlation with MVO when assessed by cardiac magnetic resonance imaging (CMRI) on the third day post-STEMI in a study of patients receiving PPCI, but MBG had no correlation with CMR-derived evaluation of MVO [78].

### 5.2. Modern Diagnostic Tools for No-Reflow Phenomenon Detection

The clinical status of patients with NR phenomenon has improved lately due to advances in medical imaging technologies that have had a significant impact on diagnostic assessment. The methods available for establishing the diagnosis of NR include cardiac magnetic resonance imaging (CMRI), myocardial contrast echocardiography (MCE), single photon emission computed tomography (SPECT), and positron emission tomography (PET).

#### 5.2.1. CMRI

The gold standard for assessing and diagnosing NR phenomenon is CMRI. Regardless of the extent of the infarct, an increased degree of MVO observed by CMRI predicts a worsened outcome in terms of LV remodeling, mortality, and heart failure hospitalizations. The extent of MVO measured by CMRI several days after PPCI is associated with heart failure hospitalizations and mortality after 12 months of the infarction [79,80].

Late gadolinium enhancement (LGE) and first pass perfusion are two gadolinium-based approaches for detecting MVO. Both procedures involve the injection of gadolinium, an extracellular substance that rapidly extravasates into the interstitial space. Gadolinium clearance in normal tissue is relatively quick (1–2 min), whereas clearance from infarcted tissue is significantly slower (approximately 30 min). In the late contrast enhancement procedure, imaging is performed 10–15 min after intravenous gadolinium administration. As a result, relative to normal cardiac tissue, infarcted myocardium appears hyper-enhanced or “bright”, while MVO shows a central hypo-enhanced zone within the hyper-enhanced area. This is known as ‘late MVO’, and it is caused by severe damage to the microvasculature, which prevents gadolinium from accessing the area [25,81].

The first pass perfusion technique is another gadolinium-based approach for MVO detection. For the first 50 heart beats, first-pass perfusion imaging is performed simultaneously with contrast injection, producing at least three short axis slices. Shortly after contrast injection, normal and infarcted myocardium show a uniform increase in signal intensity, whereas MVO appears as a lowered signal intensity (hypo-enhancement) in the core structure of the infarct persisting for more than 2 min. This is referred to as “early MVO”. The superiority of one methodology over another for quantifying MVO is still a point of contention. Several experimental studies observed certain differences in sensitivity between early and late MVO [56,82].

Late MVO, on the other hand, is considered less sensitive than early MVO because small ‘no-reflow’ areas are rapidly increased due to extracellular contrast medium diffusion from surrounding regions with normal microvasculature. As a consequence, late MVO detection may underestimate the degree of MVO [82].

Early MVO, however, had shortcomings such as insufficient LV coverage, low signal-to-noise ratio, and low spatial resolution. An imaging method that integrates early imaging with total LV coverage was proposed in an experimental study [83].

The medical evidence showed that the presence of MVO, rather than its extent, is the most accurate indicator of changes in global functionality and LV end-systolic volume at follow-up [56].

CMRI is frequently performed within the first week (day 3–5) of an AMI [84,85]. An experimental study assessed the timing of NR evaluation by CMRI. During the first week following an AMI, significant changes in LVEF, infarct size, MVO, and the extent of myocardial oedema were observed. The degree of MVO on LGE imaging decreased considerably between days 2 and 7. According to the current medical literature, the recommended time to perform CMRI for detecting NR is between days 3 and 7 [86].

#### 5.2.2. MCE

Gas-filled microbubbles are used in MCE, which are particularly effective at scattering ultrasonography. These microbubbles are small (maximum 5 μm) and do not obstruct capillaries. They can be used to detect microvascular perfusion, myocardial blood volume and capillary flow, or to identify molecular events occurring on the microvascular endothelium or within the cellular components of blood present within the microvasculature, even though they remain entirely within the vascular space [87].

Microbubbles are used to assess microvascular perfusion because their intravascular rheology matches that of erythrocytes. Microbubbles are introduced as a continuous infusion, and steady state is reached after 2–3 min, when their concentration in any blood pool, such as the LV cavity or myocardium, is constant and proportional to the blood volume concentration of that pool. When the acoustic intensity obtained from the myocardia after background subtraction is normalized to the LV cavity, the myocardial blood volume fraction is determined. Because capillary blood accounts for 90% of myocardial blood volume fraction at end-systole, a single end-systolic MCE image can be used to measure capillary density in various myocardial areas [88].

Two semi-quantitative assessment procedures were used. Firstly, a perfusion score was assigned, ranging from 1 to 3, based on the change in the myocardial signal intensity throughout the replenishment curve as well as the degree of opacification at peak contrast effect. Secondly, in each apical view, the endocardial length of the transmural contrast defect (score = 3) was quantified, averaged, and reported as a percentage of the LV endocardial length. Despite the achievement of epicardial patency, persistent contrast deficiencies were linked to MVO [89,90,91].

Low flow through an open infarct-related artery may also be evaluated using the microbubble destruction technique, in the same way as collateral flow. Lower amounts of anterograde flow lead to a slower filling of myocardium compared with normal conditions. If the capillary blood volume is basically intact (NR except in a small zone) and the myocardium fills up in less than 15–20 s on MCE, the myocardium is viable, and revascularization will improve its functionality. These results have been confirmed and validated in a clinical investigation of AMI patients [89,92].

PPCI was successful in 100 patients diagnosed with a first STEMI and single-vessel illness. The regional contrast score index, TIMI, CTFC, TMPG, and MBG were all assessed. On MCE, 168 of the 717 asynergic segments showed a lack of perfusion. Regional contrast score index was significantly linked with TMPG and CTFC (*p* = 0.031 and *p* = 0.027, respectively) but had no correlation with MBG (*p* = 0.067). There were considerably more segments with a perfusion deficiency on MCE among individuals with anterior AMI than in patients with inferior AMI (*p* = 0.0001). Angiographic methods of perfusion evaluation, such as TMPG and CTFC, correlated with MCE results. A larger amount of perfusion deficiency was associated with anterior AMI. MCE results were significantly linked to systolic LV function recovery and clinical conditions at six-months follow-up [93].

The evaluation of regional function and myocardial perfusion using MCE has been proven in experimental investigations to be superior to the TIMI score for diagnosis and prognosis in patients presenting to the emergency department with chest pain and a nondiagnostic ECG [94].

A medical investigation involving 1017 patients that were examined for chest pain in the emergency department demonstrated the incremental value of evaluating regional function and myocardial perfusion with MCE. MCE can identify the risk area, validate reperfusion success, and determine the extent of the residual infarct in AMI via the no-reflow phenomenon. Moreover, it can also be used to determine the presence and the extent of collateral perfusion after acute coronary blockage, and the mechanism that can affect myocardial viability [95].

#### 5.2.3. SPECT

SPECT is a highly accurate diagnostic tool using gamma rays, with applications for the NR phenomenon. A study showed that after PCI, 73 patients received a 201 thallous chloride (TlCl)/123 I-beta-methyl iodophenyl pentadecanoic acid (I-BMIPP) SPECT scan within one week of initial AMI. On each SPECT image, LV myocardium was separated into 17 segments and the accumulation of tracer in each segment was scored using a five-point scoring system based on American Heart Association criteria. Summing the values for all 17 segments generated the total severity score. The mismatch ratio between myocardial perfusion and metabolism was calculated using the total severity scores of 201TlCl and 123I-BMIPP. The patients enrolled in the study were classified according to TIMI flow grade. The dual-isotope myocardial SPECT of 201TlCl/123I-BMIPP indicated the biochemical degree of the NR phenomenon, whereas coronary angiography had shown clearly recanalized vascular flow. Therefore, myocardial dual-isotope SPECT may be beneficial in evaluating reperfusion treatment [96].

123I-BMIPP was used to visualize myocardial fatty acid metabolism, reflecting the memory at the onset of MI. The discrepancy between blood flow and metabolism after PCI indicates the volume of reperfused myocardium and enables severe myocardial damage without decreased flow (i.e., NR phenomenon) to be identified. Therefore, to evaluate the usefulness of 201TlCl/123 I-BMIPP dual-isotope myocardial SPECT for identifying NR, the relationship between perfusion metabolism mismatch and the degree of reflow after PCI were investigated [97]. The “no-reflow phenomenon” was originally identified during an animal experiment conducted by Kloner et al. in 1974 [98], in which the coronary artery was occluded for 90 min and then reperfusion therapy was started. The myocardial blood flow in the inner half of the damaged myocardium was only slightly improved, although the occlusion of an epicardial coronary artery was relieved. In an experimental study of human coronary arteries conducted by Schofer et al. based on nuclear cardiology, myocardial reperfusion immediately after intracoronary thrombolysis was estimated by performing intracoronary scintigraphy with technetium microalbumin aggregates before and after coronary intervention; the absence of technetium uptake was considered to reflect a lack of capillary reperfusion [50].

Planar images are utilized to develop a three-dimensional representation of myocardial perfusion in SPECT, a method which is more frequently applied and available in clinical practice today. SPECT can collect successive slices of normal and pathological areas without overlap, and with better resolution than planar imaging. SPECT imaging has been validated for the identification of coronary artery disease in several large-scale investigations; nonetheless, there are certain limitations to this imaging method. These include abnormalities generated by motion, attenuation, or extracardiac activity, all of which have an impact on image quality and reader variability. Furthermore, SPECT imaging commonly employs technetium-99m (Tc-99m) tracers, which have a poor first-pass extraction rate, resulting in an underestimate of ischemia alterations in terms of extent and severity [99,100].

SPECT imaging can be used to assess myocardial perfusion and tissue viability using a variety of methods. There is no single procedure that is appropriate for all patients, and investigations must be adjusted for each patient based on the diagnostic information required by the clinician and the patient’s characteristics. Tc-99m and thallium-201 (Tl-201) are the most frequently used radiotracers in SPECT imaging [101,102].

Images are captured in a variety of ways, including short axis cuts, long vertical and horizontal axes, and transaxial views. Poor myocardial perfusion results in lower radiotracer uptake, whereas adequate perfusion results in brighter images. The comparison of rest and stress images can contribute to identifying regions of viable, reversible perfusion abnormalities from non-reversible perfusion abnormalities. This visualization includes determining whether revascularization is required [103].

The sensitivity and specificity of SPECT perfusion imaging for the identification of coronary artery disease have been addressed in several studies. The reported sensitivity has generally varied from 70–90%, and the reported specificity has generally ranged from 60–90% [104,105].

#### 5.2.4. PET

PET is a non-invasive procedure that has a wide range of clinical and research applications in the evaluation of the pathophysiology of a variety of disorders, including neurodegenerative disorders, infections, epilepsy, seizures, psychiatric disorders, and brain tumors. It is also useful for studying blood flow. PET is a very sensitive imaging technology that allows three-dimensional mapping of positron-emitting radiopharmaceuticals in mcg doses without generating any substantial physiologic or pharmacologic effects. In contrast to MRI and computed tomography (CT), which are better suited to evaluating a body’s structure, PET is a molecular imaging technology that allows the study of biologic function in conditions of health and disease [106].

After PET examination, despite the existence of sustained TIMI flow grade 3 in the infarct-associated epicardial coronary artery, more than a third of patients were found to have reduced myocardial tissue perfusion [107].

A prospective study was conducted on 45 patients with STEMI who were treated with thrombolysis or PCI, ^13^NH_3_ being used to monitor myocardial blood flow. The areas with a myocardial blood flow <80% of regularly perfused myocardium (hypoperfusion) and <50% (NR) were automatically identified for each subject [108,109].

A whole-body PET was used for the NH_3_ (ammonia) experiments [110]. After injecting 740 million Becquerels (MBq) of ^13^NH_3_, an emission scan was taken. The perfusion study’s summed frame was generated. The myocardial image was resampled and segmented into 16 radial slices. Each polar map was divided into 33 areas, consisting of four rings of eight regions and one apex region. Using a previously established approach, a zone of normal tracer uptake was automatically delineated on the ^13^NH_3_ polar map. The reference value (100% ^13^NH_3_ uptake) was set at the mean values of that region, and the entire polar map was normalized to this value. Depending on the coronary structure shown on the angiography, the infarct-related region in each patient was manually developed on the polar map by grouping some of the 33 regions. The amount of perfusion defect in the LV was calculated by adding the myocardial wall volumes of the pixels in the affected area, and expressed as a percentage of total LV volume [108].

No association was detected between the relative resolution of ST segment deviation and any of the PET myocardial damage indicators. This fact indicates that relative resolution makes it impossible to draw clear conclusions about the magnitude of an infarct in a specific patient. In large patient populations, however, the average relative resolution of ST segment deviation has been shown to have significant predictive potential [111].

The use of PET allows the assessment of the infarct myocardium’s metabolic status and is the gold standard for determining myocardial viability. However, maintenance costs and other local considerations limit the number of institutions that may install and use PET [112].

### 5.3. Classical Versus Modern Diagnostics Methods of the No-Reflow Phenomenon

The most important characteristics of classical and modern methods for diagnosis of NR are summarized in Table 2.

## 6. Conclusions

NR has several triggers and is a complex multifactorial phenomenon. Strategies involving rapid and accurate diagnosis allow intracoronary treatment initiation at the time of PCI. This can dramatically change the prognosis of patients diagnosed with reversible NR leading to similar outcomes as for subjects without this condition. The presence of persistent NR is considered a negative prognostic factor on both the short and long term. Many diagnostic tools to identify the NR phenomenon are available, each of them with specific advantages and certain limitations. The most effective approaches among the non-invasive techniques are the assessment of microvascular flow by means of PET or Cadmium-Zinc-Telluride gated SPECT myocardial perfusion imaging. In the same study these two techniques can simultaneously evaluate: motility, thickening, perfusion, myocardial flow, myocardial flow reserve, and synchrony. In current clinical practice, invasive coronary angiography remains the gold standard technique for NR evaluation.

## Figures and Tables

**Table 1:** The incidence rate ratio of the no-reflow phenomenon.

Patients (No.)			Diagnostic Method	Incidence %	Ref.
STEMI	No-Reflow	Normal Flow			
126	47	79	MCE	37	Ito et al., 1996 [16]
1658	491	1167	PCI	42	Yang et al., 2020 [17]
5997	128	5869		2.1	Cenko et al., 2016 [18]
203	38	165		18.7	Li et al., 2018 [19]
291,380	6553	284,827		2.3	Harrison et al., 2013 [5]
93	28	65		30	Morishima et al.,1995 [20]
126	47	79		37	Ito et al., 1996 [16]
143	24	119		13.9	Rossington et al., 2020 [21]
733	54	679		16.1	Liang et al., 2017 [22]
347	110	237		32	Rezkalla et al., 2010 [23]
100	27	73		2.7	Mahmoud et al., 2019 [24]
44	11	33	MRI	25	Wu et al., 1998 [25]

STEMI, Segment elevation myocardial infarction; MCE, Myocardial contrast echocardiography; PCI, Percutaneous coronary intervention; MRI, Magnetic resonance imaging.

**Table 2:** Diagnostic methods of the no-reflow phenomenon.

Diagnostic Methods	Characteristic, Observations	Ref.
**Classical**		
ECG	Definition of the NR phenomenon: Persistence of the ST segment after perfusion of the culprit coronary artery disease by thrombolysis or coronary angiography. ST segment resolution recorded at 1 h after PCI is expected to exceed 70%. An ST segment resolution less than 70% at 1 h is a marker of NR. A successful myocardial reperfusion involves rapid ST segment resolution, parameter very specific (91%), sensitivity moderate (77%).	Caiazzo et al., 2020, Tatu-Chițoiu et al., 2014 [113,114]
Coronary angiography	Enhances semiquantitative assessment of the epicardial coronary blood flow. A suboptimal TIMI flow grade and blush grade, as well as a prolonged “TIMI frame count” indicate the detection of NR. Even if the sensitivity of TIMI flow evaluation is modest, corroboration between MBG grade and ST segment evaluation has a prognostic impact independent of the revascularization method, an aspect that requires accurate assessment of the two elements. MBG 2 to 3 is associated with a rapid ST segment resolution >70%, correlated with a favorable prognosis. CTFC is the summed frame count from the interventional opening of the coronary artery responsible for the MI to the crossing of the contrast substance distally post-revascularization. CTFC is considered the “gold standard” for NR evaluation. Routine assessment of anterior descending artery CTFC compared to the other epicardial coronary arteries is required due to its importance and size. An extended frame count above 20 is a criterion for the detection of NR.	Gupta et al., 2016 Sorraja et al., 2005 Thygesen et al., 2018 Khan et al., 2020 Seyfeli et al., 2007 Bauer et al., 2020 Kaya et al., 2007 Giugliano et al., 2004 [27,74,115,116,117,118,119,120]
**Modern**		
MCE	Absence or poor presence of the contrast substance administered during myocardial echocardiography indicates NR. We should assess the success of post-STEMI myocardial reperfusion compared to coronary angiography.	Kaul et al., 2006 [121]
Intracardiac Echocardiography	The reversal of systolic flow, anterograde reduction of systolic flow, and diastolic flow with a rapidly decelerating slope defines the NR phenomenon. Post PPCI patients with NR due to distal microembolization will present slow intracoronary flow velocity throughout the cardiac cycle.	Ramjane et al., 2008 [4]
CMRI	Provides highly accurate assessment of myocardial tissue. Detects the presence and extension of the infarcted area, tissue oedema present in the infarct area, and MVO as the expression of the NR phenomenon. First contrast agent injection illustrates myocardial perfusion, while delayed contrast myocardial MRI reveals myocardial necrosis. High spatial resolution allows assessment of the transmural extent of the NR, as well as the infarcted area.	Wu et al., 2012 [122]
SPECT	Good accuracy in assessing the degree of myocardial flow, as well as the presence of vascular micro-obstruction. Reproduces the degree of NR. NR appears as vascular micro-obstruction characterized by hypo-amplification due to reduced blood flow.	Shimizu et al., 2006 [96]
PET	Highly accurate for NR detection. Non-invasive diagnostic technique without major adverse effects. Shows in detail the hypoxic myocardial territory but it is less used in current practice due to the high cost.	Jeremy et al., 1990 [123]

ECG, electrocardiogram; TIMI, thrombolysis in myocardial infarction; MBG, myocardial blush grade; CTFC, corrected TIMI frame count; MCE, myocardial contrast echocardiography; STEMI, segment elevation myocardial infarction; PCI, percutaneous coronary intervention; CMRI, cardiac magnetic resonance imaging; SPECT, single photon emission computed tomography; PET, positron emission tomography, NR, no-reflow; MVO, microvascular obstruction.

## References

[1] Ito H. (2006). No-reflow phenomenon and prognosis in patients with acute myocardial infarction. Nat. Clin. Pract. Cardiovasc. Med..

[2] Ndrepepa G., Tiroch K., Fusaro M., Keta D., Seyfarth M., Byrne R.A., Pache J., Alger P., Mehilli J., Schömig A. (2010). 5-year prognostic value of no-reflow phenomenon after percutaneous coronary intervention in patients with acute myocardial infarction. J. Am. Coll. Cardiol..

[3] Pantea-Roșan L.R., Pantea V.A., Bungau S., Tit D.M., Behl T., Vesa C.M., Bustea C., Moleriu R.D., Rus M., Popescu M.I. (2020). No-reflow after PPCI—A predictor of short-term outcomes in STEMI patients. J. Clin. Med..

[4] Ramjane K., Lei H., Jin C. (2008). The diagnosis and treatment of the no-reflow phenomenon in patients with myocardial infarction undergoing percutaneous coronary intervention. Exp. Clin. Cardiol..

[5] Harrison R.W., Aggarwal A., Ou F.S., Klein L.W., Rumsfeld J.S., Roe M.T., Wang T.Y. (2013). Incidence and Outcomes of no-reflow phenomenon during percutaneous coronary intervention among patients with acute myocardial infarction. Am. J. Cardiol..

[6] Barletta G., Bene M.R. (2015). Del Myocardial perfusion echocardiography and coronary microvascular dysfunction. World J. Cardiol..

[7] Allencherril J., Jneid H., Atar D., Alam M., Levine G., Kloner R.A., Birnbaum Y. (2019). Pathophysiology, Diagnosis, and management of the no-reflow phenomenon. Cardiovasc. Drugs Ther..

[8] Kaur G., Baghdasaryan P., Natarajan B., Sethi P., Mukherjee A., Varadarajan P., Pai R.G. (2021). Pathophysiology, diagnosis, and management of coronary no-reflow phenomenon. Int. J. Angiol..

[9] Namazi M., Mahmoudi E., Safi M., Jenab Y., Va-Kili H., Saadat H., Parsa S.A., Khaheshi I., Talasaz A.H., Hosseini S.H. (2021). The No-reflow phenomenon: Is it predictable by demographic factors and routine laboratory data?. Acta Biomed..

[10] Gheorghe G., Toth P.P., Bungau S., Behl T., Ilie M., Stoian A.P., Bratu O.G., Bacalbasa N., Rus M., Diaconu C.C. (2020). Cardiovascular risk and statin therapy considerations in women. Diagnostics.

[11] Porto I., Ashar V., Mitchell A. (2006). Pharmacological management of no reflow during percutaneous coronary intervention. Curr. Vasc. Pharmacol..

[12] Behl T., Bungau S., Kumar K., Zengin G., Khan F., Kumar A., Kaur R., Venkatachalam T., Tit D.M., Vesa C.M. (2020). Pleotropic effects of polyphenols in cardiovascular system. Biomed. Pharmacother..

[13] Sabir F., Barani M., Mukhtar M., Rahdar A., Cucchiarini M., Zafar M.N., Behl T., Bungau S. (2021). Nanodiagnosis and nanotreatment of cardiovascular diseases: An overview. Chemosensors.

[14] Page M.J., McKenzie J.E., Bossuyt P.M., Boutron I., Hoffmann T.C., Mulrow C.D., Shamseer L., Tetzlaff J.M., Akl E.A., Brennan S.E. (2021). The PRISMA 2020 statement: An updated guideline for reporting systematic reviews. BMJ.

[15] Mandurino-Mirizzi A., Spolverini M., Attanasio A., Crimi G. (2020). Management of no-reflow: Still an unsolved problem?. J. Phlebol. Lymphology.

[16] Ito H., Maruyama A., Iwakura K., Takiuchi S., Masuyama T., Hori M., Higashino Y., Fujii K., Minamino T. (1996). Clinical implications of the ‘no reflow’ phenomenon. Circulation.

[17] Yang L., Cong H., Lu Y., Chen X., Liu Y. (2020). Prediction of no-reflow phenomenon in patients treated with primary percutaneous coronary intervention for ST-segment elevation myocardial infarction. Medicine.

[18] Cenko E., Ricci B., Kedev S., Kalpak O., Câlmâc L., Vasiljevic Z., Knežević B., Dilic M., Miličić D., Manfrini O. (2016). The no-reflow phenomenon in the young and in the elderly. Int. J. Cardiol..

[19] Li H., Fu D.G., Liu F.Y., Zhou H., Li X.M. (2018). Evaluation of related factors, prediction and treatment drugs of no-reflow phenomenon in patients with acute ST-segment elevation myocardial infarction after direct PCI. Exp. Ther. Med..

[20] Morishima I., Sone T., Mokuno S., Taga S., Shimauchi A., Oki Y., Kondo J., Tsuboi H., Sassa H. (1995). Clinical significance of no-reflow phenomenon observed on angiography after successful treatment of acute myocardial infarction with percutaneous transluminal coronary angioplasty. Am. Heart J..

[21] Rossington J.A., Sol E., Masoura K., Aznaouridis K., Chelliah R., Cunnington M., Davison B., John J., Oliver R., Hoye A. (2020). No-reflow phenomenon and comparison to the normal-flow population postprimary percutaneous coronary intervention for ST elevation myocardial infarction: Case-control study (NORM PPCI). Open Heart.

[22] Liang T., Liu M., Wu C., Zhang Q., Lu L., Wang Z. (2017). Risk factors for no-reflow phenomenon after percutaneous coronary intervention in patients with acute coronary syndrome. Rev. Investig. Clin..

[23] Rezkalla S.H., Dharmashankar K.C., Abdalrahman I.B., Kloner R.A. (2010). No-reflow phenomenon following percutaneous coronary intervention for acute myocardial infarction: Incidence, outcome, and effect of pharmacologic therapy. J. Interv. Cardiol..

[24] Mahmoud A.H., Taha N.M., Baraka K., Ashraf M., Shehata S. (2019). Clinical and procedural predictors of suboptimal myocardial reperfusion in primary percutaneous coronary intervention. IJC Heart Vasc..

[25] Wu K.C., Zerhouni E.A., Judd R.M., Lugo-Olivieri C.H., Barouch L.A., Schulman S.P., Blumenthal R.S., Lima J.A.C. (1998). Prognostic significance of microvascular obstruction by magnetic resonance imaging in patients with acute myocardial infarction. Circulation.

[26] Oikonomou E., Mourouzis K., Vogiatzi G., Siasos G., Deftereos S., Papaioannou S., Latsios G., Tsalamandris S., Tousoulis D. (2018). Coronary microcirculation and the no-reflow phenomenon. Curr. Pharm. Des..

[27] Gupta S., Gupta M.M. (2016). No reflow phenomenon in percutaneous coronary interventions in ST-segment elevation myocardial infarction. Indian Heart J..

[28] Jaffe R., Charron T., Puley G., Dick A., Strauss B.H. (2008). Microvascular obstruction and the no-reflow phenomenon after percutaneous coronary intervention. Circulation.

[29] Katayama Y., Taruya A., Kashiwagi M., Ozaki Y., Shiono Y., Tanimoto T., Yoshikawa T., Kondo T., Tanaka A. (2022). No-reflow phenomenon and in vivo cholesterol crystals combined with lipid core in acute myocardial infarction. Int. J. Cardiol. Heart Vasc..

[30] Niccoli G., Scalone G., Lerman A., Crea F. (2016). Coronary microvascular obstruction in acute myocardial infarction. Eur. Heart J..

[31] Patel K.K., Spertus J.A., Chan P.S., Sperry B.W., Thompson R.C., Al Badarin F., Kennedy K.F., Case J.A., Courter S., Saeed I.M. (2019). Extent of myocardial ischemia on positron emission tomography and survival benefit with early revascularization. J. Am. Coll. Cardiol..

[32] Minicucci M.F., Azevedo P.S., Polegato B.F., Paiva S.A.R., Zornoff L.A.M. (2011). Heart failure after myocardial infarction: Clinical implications and treatment. Clin. Cardiol..

[33] Yellon D.M., Hausenloy D.J. (2007). Myocardial reperfusion injury. N. Engl. J. Med..

[34] Bekkers S.C.A.M., Yazdani S.K., Virmani R., Waltenberger J. (2010). Microvascular obstruction: Underlying pathophysiology and clinical diagnosis. J. Am. Coll. Cardiol..

[35] Vrints C.J.M. (2009). Pathophysiology of the no-reflow phenomenon. Acute Card. Care.

[36] Fajar J.K., Heriansyah T., Rohman M.S. (2018). The predictors of no reflow phenomenon after percutaneous coronary intervention in patients with ST elevation myocardial infarction: A meta-analysis. Indian Heart J..

[37] Shankar S.S., Steinberg H.O. (2005). Obesity and endothelial dysfunction. Semin. Vasc. Med..

[38] Okutsu M., Mitomo S., Nakamura S., Nakamura S. (2021). Interventional cardiology are the high-risk plaques of no-reflow phenomenon equivalent to vulnerable plaques?. Interv. Cardiol..

[39] Hong Y.J., Jeong M.H., Choi Y.H., Ko J.S., Lee M.G., Kang W.Y., Lee S.E., Kim S.H., Park K.H., Sim D.S. (2011). Impact of plaque components on no-reflow phenomenon after stent deployment in patients with acute coronary syndrome: A virtual histology-intravascular ultrasound analysis. Eur. Heart J..

[40] Obaid D.R., Calvert P.A., Gopalan D., Parker R.A., Hoole S.P., West N.E.J., Goddard M., Rudd J.H.F., Bennett M.R. (2013). Atherosclerotic plaque composition and classification identified by coronary computed tomography: Assessment of computed tomography-generated plaque maps compared with virtual histology intravascular ultrasound and histology. Circ. Cardiovasc. Imaging.

[41] Shioi A., Ikari Y. (2018). Plaque calcification during atherosclerosis progression and regression. J. Atheroscler. Thromb..

[42] Gonçalves I., den Ruijter H., Nahrendorf M., Pasterkamp G. (2015). Detecting the vulnerable plaque in patients. J. Intern. Med..

[43] Katakami N. (2018). Mechanism of development of atherosclerosis and cardiovascular disease in diabetes mellitus. J. Atheroscler. Thromb..

[44] Fox C.S., Golden S.H., Anderson C., Bray G.A., Burke L.E., De Boer I.H., Deedwania P., Eckel R.H., Ershow A.G., Fradkin J. (2015). Update on prevention of cardiovascular disease in adults with type 2 diabetes mellitus in light of recent evidence: A scientific statement from the American heart association and the American diabetes association. Diabetes Care.

[45] Bolad I., Delafontaine P. (2005). Endothelial dysfunction: Its role in hypertensive coronary disease. Curr. Opin. Cardiol..

[46] Tasar O., Karabay A.K., Oduncu V., Kirma C. (2019). Predictors and outcomes of no-reflow phenomenon in patients with acute ST-segment elevation myocardial infarction undergoing primary percutaneous coronary intervention. Coron. Artery Dis..

[47] Wang Y., Zhao H.W., Wang C.F., Zhang X.J., Tao J., Cui C.S., Meng Q.K., Zhu Y., Luo D.F., Hou A.J. (2020). Incidence, predictors, and prognosis of coronary slow-flow and no-reflow phenomenon in patients with chronic total occlusion who underwent percutaneous coronary intervention. Ther. Clin. Risk Manag..

[48] Reffelmann T., Kloner R.A. (2002). The “no-reflow” phenomenon: Basic science and clinical correlates. Heart.

[49] Bouleti C., Mewton N., Germain S. (2015). The no-reflow phenomenon: State of the art. Arch. Cardiovasc. Dis..

[50] Schofer J., Montz R., Mathey D.G. (1985). Scintigraphic evidence of the “No reflow” phenomenon in human beings after coronary thrombolysis. J. Am. Coll. Cardiol..

[51] Porter T.R., Li S., Oster R., Deligonul U. (1998). The clinical implications of no reflow demonstrated with intravenous perfluorocarbon containing microbubbles following restoration of Thrombolysis In Myocardial Infarction (TIMI) 3 flow in patients with acute myocardial infarction. Am. J. Cardiol..

[52] Birnbaum Y., Nikus K., Kligfield P., Fiol M., Barrabés J.A., Sionis A., Pahlm O., Garcia Niebla J., Bayès De Luna A. (2014). The role of the ECG in diagnosis, risk estimation, and catheterization laboratory activation in patients with acute coronary syndromes: A consensus document. Ann. Noninvasive Electrocardiol..

[53] Ding S., Zhao H., Qiao Z.Q., Yang F., Wang W., Gao L.C., Kong L.C., Xu R.-D., Ge H., Shen X.D. (2015). Early resolution of ST-segment elevation after reperfusion therapy for acute myocardial infarction: Its relation to echocardiography-determined left ventricular global and regional function and deformation. J. Electrocardiol..

[54] Van’t Hof A.W.J., Liem A., De Boer M.J., Zijlstra F. (1997). Clinical value of 12-lead electrocardiogram after successful reperfusion therapy for acute myocardial infarction. Lancet.

[55] Haager P.K., Christott P., Heussen N., Lepper W., Hanrath P., Hoffmann R. (2003). Prediction of clinical outcome after mechanical revascularization in acute myocardial infarction by markers of myocardial reperfusion. J. Am. Coll. Cardiol..

[56] Nijveldt R., Beek A.M., Hirsch A., Stoel M.G., Hofman M.B.M., Umans V.A.W.M., Algra P.R., Twisk J.W.R., van Rossum A.C. (2008). Functional recovery after acute myocardial infarction: Comparison between angiography, electrocardiography, and cardiovascular magnetic resonance measures of microvascular injury. J. Am. Coll. Cardiol..

[57] Santoro G.M., Valenti R., Buonamici P., Bolognese L., Cerisano G., Moschi G., Trapani M., Antoniucci D., Fazzini P.F. (1998). Relation between ST-segment changes and myocardial perfusion evaluated by myocardial contrast echocardiography in patients with acute myocardial infarction treated with direct angioplasty. Am. J. Cardiol..

[58] Wehrens X.H.T., Doevendans P.A., Oude Ophuis T.J., Wellens H.J.J. (2000). A comparison of electrocardiographic changes during reperfusion of acute myocardial infarction by thrombolysis or percutaneous transluminal coronary angioplasty. Am. Heart J..

[59] Chesebro J.H., Knatterud G., Roberts R., Borer J., Cohen L.S., Dalen J., Dodge H.T., Francis C.K., Hillis D., Ludbrook P. (1987). Thrombolysis in Myocardial Infarction (TIMI) Trial, Phase I: A comparison between intravenous tissue plasminogen activator and intravenous streptokinase. Clinical findings through hospital discharge. Circulation.

[60] Ndrepepa G., Tiroch K., Keta D., Fusaro M., Seyfarth M., Pache J., Mehilli J., Schömig A., Kastrati A. (2010). Predictive factors and impact of no reflow after primary percutaneous coronary intervention in patients with acute myocardial infarction. Circ. Cardiovasc. Interv..

[61] Grygier M., Araszkiewicz A., Lesiak M., Janus M., Kowal J., Skorupski W., Pyda M., Mitkowski P., Grajek S. (2011). New method of intracoronary adenosine injection to prevent microvascular reperfusion injury in patients with acute myocardial infarction undergoing percutaneous coronary intervention. Am. J. Cardiol..

[62] Niccoli G., Giubilato S., Russo E., Spaziani C., Leo A., Porto I., Leone A.M., Burzotta F., Riondino S., Pulcinelli F. (2008). Plasma levels of thromboxane A2 on admission are associated with no-reflow after primary percutaneous coronary intervention. Eur. Heart J..

[63] Niccoli G., Lanza G.A., Shaw S., Romagnoli E., Gioia D., Burzotta F., Trani C., Mazzari M.A., Mongiardo R., De Vita M. (2006). Endothelin-1 and acute myocardial infarction: A no-reflow mediator after successful percutaneous myocardial revascularization. Eur. Heart J..

[64] Vogt A., von Essen R., Tebbe U., Feuerer W., Appel K.F., Niederer W., Neuhaus K.L. (1994). Frequency of achieving optimal reperfusion with thrombolysis in acute myocardial infarction (analysis of four German multicenter studies). Am. J. Cardiol..

[65] Zijlstra F., de Boer M.J., Hoorntje J., Reiffers S., Reiber J., Suryapranata H. (1993). A comparison of immediate coronary angioplasty with intravenous streptokinase in acute myocardial infarction. N. Engl. J. Med..

[66] Simes R.J., Topol E.J., Holmes D.R., White H.D., Rutsch W.R., Vahanian A., Simoons M.L., Morris D., Betriu A., Califf R.M. (1995). Link between the angiographic substudy and mortality outcomes in a large randomized trial of myocardial reperfusion. Importance of early and complete infarct artery reperfusion. GUSTO-I investigators. Circulation.

[67] Yano A., Ito H., Iwakura K., Kimura R., Tanaka K., Okamura A., Kawano S., Masuyama T., Fujii K. (2004). Myocardial contrast echocardiography with a new calibration method can estimate myocardial viabilityin patients with myocardial infarction. J. Am. Coll. Cardiol..

[68] Karagounis L., Sorensen S.G., Menlove R.L., Moreno F., Anderson J.L. (1992). Does thrombolysis in myocardial infarction (TIMI) perfusion grade 2 represent a mostly patent artery or a mostly occluded artery? Enzymatic and electrocardiographic evidence from the TEAM-2 study. Second Multicenter Thrombolysis Trial of Eminase in Acute Myocardial Infarction. J. Am. Coll. Cardiol..

[69] Li X., Lyu L., Yang W., Pan J., Dong M., Zhang M., Zhang P. (2021). Identification of flow-limiting coronary stenosis with PCS: A new cost-effective index derived from the product of corrected TIMI frame count and percent diameter stenosis. Front. Cardiovasc. Med..

[70] Gibson C.M., Murphy S.A., Rizzo M.J., Ryan K.A., Marble S.J., McCabe C.H., Cannon C.P., Van De Werf F., Braunwald E. (1999). Relationship between TIMI frame count and clinical outcomes after thrombolytic administration. Thrombolysis in Myocardial Infarction (TIMI) study group. Circulation.

[71] Yen C.H., Yen H.I., Hou C.J.Y., Chou Y.S., Tsai C.H. (2007). Thrombolysis in Myocardial Infarction frame count in single-vessel disease after angioplasty. Int. J. Gerontol..

[72] Van’t Hof A.W.J., Liem A., Suryapranata H., Hoorntje J.C.A., De Boer M.J., Zijlstra F. (1998). Angiographic assessment of myocardial reperfusion in patients treated with primary angioplasty for acute myocardial infarction: Myocardial blush grade. Zwolle Myocardial infarction study group. Circulation.

[73] Kampinga M.A., Nijsten M.W.N., Gu Y.L., Dijk W.A., De Smet B.J.G.L., Van Den Heuvel A.F.M., Tan E.S., Zijlstra F. (2010). Is the myocardial blush grade scored by the operator during primary percutaneous coronary intervention of prognostic value in patients with ST-elevation myocardial infarction in routine clinical practice?. Circ. Cardiovasc. Interv..

[74] Sorajja P., Gersh B.J., Costantini C., McLaughlin M.G., Zimetbaum P., Cox D.A., Garcia E., Tcheng J.E., Mehran R., Lansky A.J. (2005). Combined prognostic utility of ST-segment recovery and myocardial blush after primary percutaneous coronary intervention in acute myocardial infarction. Eur. Heart J..

[75] Gibson C.M., Cannon C.P., Murphy S.A., Ryan K.A., Mesley R., Marble S.J., McCabe C.H., Van De Werf F., Braunwald E. (2000). Relationship of TIMI myocardial perfusion grade to mortality after administration of thrombolytic drugs. Circulation.

[76] Gibson C.M., Cannon C.P., Murphy S.A., Marble S.J., Barron H.V., Braunwald E. (2002). Relationship of the TIMI myocardial perfusion grades, flow grades, frame count, and percutaneous coronary intervention to long-term outcomes after thrombolytic administration in acute myocardial infarction. Circulation.

[77] Carrick D., Oldroyd K.G., McEntegart M., Haig C., Petrie M.C., Eteiba H., Hood S., Owens C., Watkins S., Layland J. (2014). A randomized trial of deferred stenting versus immediate stenting to prevent no- or slow-reflow in acute ST-segment elevation myocardial infarction (DEFER-STEMI). J. Am. Coll. Cardiol..

[78] Wong D.T.L., Leung M.C.H., Richardson J.D., Puri R., Bertaso A.G., Williams K., Meredith I.T., Teo K.S.L., Worthley M.I., Worthley S.G. (2012). Cardiac magnetic resonance derived late microvascular obstruction assessment post ST-segment elevation myocardial infarction is the best predictor of left ventricular function: A comparison of angiographic and cardiac magnetic resonance derived measurements. Int. J. Cardiovasc. Imaging.

[79] De Waha S., Patel M.R., Granger C.B., Ohman E.M., Maehara A., Eitel I., Ben-Yehuda O., Jenkins P., Thiele H., Stone G.W. (2017). Relationship between microvascular obstruction and adverse events following primary percutaneous coronary intervention for ST-segment elevation myocardial infarction: An individual patient data pooled analysis from seven randomized trials. Eur. Heart J..

[80] Bolognese L., Carrabba N., Parodi G., Santoro G.M., Buonamici P., Cerisano G., Antoniucci D. (2004). Impact of microvascular dysfunction on left ventricular remodeling and long-term clinical outcome after primary coronary angioplasty for acute myocardial infarction. Circulation.

[81] Wu K.C., Kim R.J., Bluemke D.A., Rochitte C.E., Zerhouni E.A., Becker L.C., Lima J.A.C. (1998). Quantification and time course of microvascular obstruction by contrast-enhanced echocardiography and magnetic resonance imaging following acute myocardial infarction and reperfusion. J. Am. Coll. Cardiol..

[82] Lund G.K., Stork A., Saeed M., Bansmann M.P., Gerken J.H., Müller V., Mester J., Higgins C.B., Adam G., Meinertz T. (2004). Acute myocardial infarction: Evaluation with first-pass enhancement and delayed enhancement MR imaging compared with 201Tl SPECT imaging. Radiology.

[83] Bekkers S.C.A.M., Backes W.H., Kim R.J., Snoep G., Gorgels A.P.M., Passos V.L., Waltenberger J., Crijns H.J.G.M., Schalla S. (2009). Detection and characteristics of microvascular obstruction in reperfused acute myocardial infarction using an optimized protocol for contrast-enhanced cardiovascular magnetic resonance imaging. Eur. Radiol..

[84] De Waha S., Desch S., Eitel I., Fuernau G., Zachrau J., Leuschner A., Gutberlet M., Schuler G., Thiele H. (2010). Impact of early vs. late microvascular obstruction assessed by magnetic resonance imaging on long-term outcome after ST-elevation myocardial infarction: A comparison with traditional prognostic markers. Eur. Heart J..

[85] Wong D.T.L., Leung M.C.H., Das R., Liew G.Y.H., Teo K.S.L., Chew D.P., Meredith I.T., Worthley M.I., Worthley S.G. (2013). Intracoronary ECG during primary percutaneous coronary intervention for ST-segment elevation myocardial infarction predicts microvascular obstruction and infarct size. Int. J. Cardiol..

[86] Mather A.N., Fairbairn T.A., Artis N.J., Greenwood J.P., Plein S. (2011). Timing of cardiovascular MR imaging after acute myocardial infarction: Effect on estimates of infarct characteristics and prediction of late ventricular remodeling. Radiology.

[87] Jayaweera A.R., Edwards N., Glasheen W.P., Villanueva F.S., Abbott R.D., Kaul S. (1994). In vivo myocardial kinetics of air-filled albumin microbubbles during myocardial contrast echocardiography. Comparison with radiolabeled red blood cells. Circ. Res..

[88] Kaul S., Villanueva F.S. (1992). Is the determination of myocardial perfusion necessary to evaluate the success of reperfusion when the infarct-related artery is open?. Circulation.

[89] Swinburn J.M.A., Lahiri A., Senior R. (2001). Intravenous myocardial contrast echocardiography predicts recovery of dysynergic myocardium early after acute myocardial infarction. J. Am. Coll. Cardiol..

[90] Agati L., Tonti G., Pedrizzetti G., Magri F., Funaro S., Madonna M., Celani F., Messager T., Broillet A. (2004). Clinical application of quantitative analysis in real-time MCE. Eur. J. Echocardiogr..

[91] Ito H., Tomooka T., Sakai N., Yu H., Higashino Y., Fujii K., Masuyama T., Kitabatake A., Minamino T. (1992). Lack of myocardial perfusion immediately after successful thrombolysis. A predictor of poor recovery of left ventricular function in anterior myocardial infarction. Circulation.

[92] Hayat S.A., Senior R. (2008). Myocardial contrast echocardiography in ST elevation myocardial infarction: Ready for prime time?. Eur. Heart J..

[93] Tomaszuk-Kazberuk A., Sobkowicz B., Kaminski K., Gugala K., Mezynski G., Dobrzycki S., Lewczuk A., Kazberuk W., Musial W.J. (2008). Myocardial perfusion assessed by contrast echocardiography correlates with angiographic perfusion parameters in patients with a first acute myocardial infarction successfully treated with angioplasty. Can. J. Cardiol..

[94] Tong K.L., Kaul S., Wang X.Q., Rinkevich D., Kalvaitis S., Belcik T., Lepper W., Foster W.A., Wei K. (2005). Myocardial contrast echocardiography versus Thrombolysis in Myocardial Infarction score in patients presenting to the emergency department with chest pain and a nondiagnostic electrocardiogram. J. Am. Coll. Cardiol..

[95] Rinkevich D., Kaul S., Wang X.Q., Khim L.T., Belcik T., Kalvaitis S., Lepper W., Dent J.M., Wei K. (2005). Regional left ventricular perfusion and function in patients presenting to the emergency department with chest pain and no ST-segment elevation. Eur. Heart J..

[96] Shimizu Y., Kumita S.I., Cho K., Toba M., Mizumura S., Tanaka K., Takano T., Kumazaki T. (2006). Evaluation of no-reflow phenomenon using 201TlCl/123I-BMIPP dual-isotope myocardial SPECT. J. Nippon Med. Sch..

[97] Hamada S., Nakamura S., Sugiura T., Murakami T., Fujimoto T., Watanabe J., Baden M., Hatada K., Iwasaka T. (1999). Early detection of the no-reflow phenomenon in reperfused acute myocardial infarction using technetium-99m tetrofosmin imaging. Eur. J. Nucl. Med..

[98] Kloner R.A., Ganote C.E., Jennings R.B. (1974). The “no-reflow” phenomenon after temporary coronary occlusion in the dog. J. Clin. Investig..

[99] Pelletier-Galarneau M., Martineau P., El Fakhri G. (2019). Quantification of PET myocardial blood flow. Curr. Cardiol. Rep..

[100] Jaarsma C., Leiner T., Bekkers S.C., Crijns H.J., Wildberger J.E., Nagel E., Nelemans P.J., Schalla S. (2012). Diagnostic performance of noninvasive myocardial perfusion imaging using single-photon emission computed tomography, cardiac magnetic resonance, and positron emission tomography imaging for the detection of obstructive coronary artery disease: A meta-analysis. J. Am. Coll. Cardiol..

[101] Akutsu Y., Kaneko K., Kodama Y., Li H.L., Nishimura H., Hamazaki Y., Suyama J., Shinozuka A., Gokan T., Kobayashi Y. (2008). Technetium-99m pyrophosphate/thallium-201 dual-isotope SPECT imaging predicts reperfusion injury in patients with acute myocardial infarction after reperfusion. Eur. J. Nucl. Med. Mol. Imaging.

[102] Henzlova M.J., Duvall W.L., Einstein A.J., Travin M.I., Verberne H.J. (2016). ASNC imaging guidelines for SPECT nuclear cardiology procedures: Stress, protocols, and tracers. J. Nucl. Cardiol..

[103] Iskandrian A.S. (1991). Single-photon emission computed tomographic thallium imaging with adenosine, dipyridamole, and exercise. Am. Heart J..

[104] Arbab-Zadeh A., Carli M.F.D., Cerci R., George R.T., Chen M.Y., Dewey M., Niinuma H., Vavere A.L., Betoko A., Plotkin M. (2015). Accuracy of CT angiography and SPECT myocardial perfusion imaging for the diagnosis of coronary artery disease. Circ. Cardiovasc. Imaging.

[105] Liu H., Wu J., Miller E.J., Liu C., Liu Y., Liu Y.H. (2021). Diagnostic accuracy of stress-only myocardial perfusion SPECT improved by deep learning. Eur. J. Nucl. Med. Mol. Imaging.

[106] Lameka K., Farwell M.D., Ichise M. (2016). Positron emission tomography. Handb. Clin. Neurol..

[107] Maes A., Van De Werf F., Nuyts J., Bormans G., Desmet W., Mortelmans L. (1995). Impaired myocardial tissue perfusion early after successful thrombolysis. Circulation.

[108] Desmet W.J., Mesotten L.V., Maes A.F., Heidbüchel H.P., Mortelmans L.A., Van De Werf F.J. (2004). Relation between different methods for analysing ST segment deviation and infarct size as assessed by positron emission tomography. Heart.

[109] Salustri A., Pozzoli M.M.A., Ilmer B., Hermans W., Reijs A.E.M., Reiber J.H.C., Roelandt J.R.T.C., Fioretti P.M. (1992). Exercise echocardiography and single photon emission computed tomography in patients with left anterior descending coronary artery stenosis. Int. J. Card. Imaging.

[110] Driessen R.S., Raijmakers P.G., Stuijfzand W.J., Knaapen P. (2017). Myocardial perfusion imaging with PET. Int. J. Cardiovasc. Imaging.

[111] Claeys M.J., Bosmans J., Veenstra L., Jorens P., De Raedt H., Vrints C.J. (1999). Determinants and prognostic implications of persistent ST-segment elevation after primary angioplasty for acute myocardial infarction importance of microvascular reperfusion injury on clinical outcome. Circulation.

[112] Tamaki N., Kawamoto M., Tadamura E., Magata Y., Yonekura Y., Nohara R., Sasayama S., Nishimura K., Ban T., Konishi J. (1995). Prediction of reversible ischemia after revascularization. Perfusion and metabolic studies with positron emission tomography. Circulation.

[113] Caiazzo G., Musci R.L., Frediani L., Umińska J., Wanha W., Filipiak K.J., Kubica J., Navarese E.P. (2020). State of the art: No-reflow phenomenon. Cardiol. Clin..

[114] Tatu-Chițoiu G. (2014). Electrocardiograma in Reperfuzia Miocardica.

[115] Giugliano R.P., Sabatine M.S., Gibson C.M., Roe M.T., Harrington R.A., Murphy S.A., Morrow D.A., Antman E.M., Braunwald E. (2004). Combined assessment of thrombolysis in myocardial infarction flow grade, myocardial perfusion grade, and ST-segment resolution to evaluate epicardial and myocardial reperfusion. Am. J. Cardiol..

[116] Thygesen K., Alpert J.S., Jaffe A.S., Chaitman B.R., Bax J.J., Morrow D.A., White H.D., Corbett S., Chettibi M., Hayrapetyan H. (2018). Fourth universal definition of myocardial infarction (2018). Circulation.

[117] Khan R., Zarak M.S., Munir U., Ahmed K., Ullah A. (2020). Thrombolysis in Myocardial Infarction (TIMI) risk score assessment for complications in acute anterior wall ST elevation myocardial infarction. Cureus.

[118] Seyfeli E., Abaci A., Kula M., Topsakal R., Eryol N.K., Arinc H., Ozdogru I., Ergin A. (2007). Myocardial blush grade: To evaluate myocardial viability in patients with acute myocardial infarction. Angiology.

[119] Bauer T., Zeymer U., Diallo A., Vicaut E., Bolognese L., Cequier A., Huber K., Montalescot G., Hamm C.W., van’t Hof A.W. (2020). Impact of preprocedural TIMI flow on clinical outcome in low-risk patients with ST-elevation myocardial infarction: Results from the ATLANTIC study. Catheter. Cardiovasc. Interv..

[120] Kaya M.G., Arslan F., Abaci A., Van Der Heijden G., Timurkay-Nak T., Cengel A. (2007). Myocardial blush grade: A predictor for major adverse cardiac events after primary PTCA with stent implantation for acute myocardial infarction. Acta Cardiol..

[121] Kaul S. (2006). Evaluating the “no reflow” phenomenon with myocardial contrast echocardiography. Basic Res. Cardiol..

[122] Wu K.C. (2012). CMR of microvascular obstruction and hemorrhage in myocardial infarction. J. Cardiovasc. Magn. Reson..

[123] Jeremy R.W., Links J.M., Becker L.C. (1990). Progressive failure of coronary flow during reperfusion of myocardial infarction: Documentation of the no reflow phenomenon with positron emission tomography. J. Am. Coll. Cardiol..

